# Supplementary material for: Tumor Burden and Health-Related Quality of Life in Patients with Melanoma In-Transit Metastases
Source: Cancers (Basel). 2022 Dec 27;15(1):161. doi: 10.3390/cancers15010161 (PMC9817978; doi:10.3390/cancers15010161)
Supplement: Supplementary file 1 [file cancers-15-00161-s001.zip › cancers-2032944-supplementary.pdf]

Supplementary Materials:

Table S1. Multiple and hierarchical ordinary least squares (OLS) regression showing the total FACT-M score regressed on patient characteristics (sex, age) in Step 1, and tumor burden (number of tumors, tumor size, localization, lymph node metastasis) in Step 2.

	Step 1					Step 2				
	B	[95% CI]		Beta	p	B	[95% CI]		Beta	p
		LL	UL				LL	UL		
Sex[a]	8.42	-.27	17.11	.21	.057	11.64	2.91	20.36	.28	.010
Age	.26	-.12	-.64	.15	.179	.25	-.14	.64	.14	.199
Number						-.40	-.64	-.17	-.37	.001
Size						-.15	-.35	.06	-.15	.164
Localization[b]						6.99	-6.28	20.26	.11	.297
Metastasis[c]						.94	-8.01	9.89	.02	.835
	F(2, 81)=2,78, p=.068; R^2=.04					F(6, 71)=3,38, p=.005; R^2=.16				

[a]Female vs. male
[b]Leg vs. arm
[c]No vs. Yes

Table S2. Multiple and hierarchical ordinary least squares (OLS) regression showing the FACT-M subscale FACT-G score regressed on patient characteristics (sex, age) in Step 1, and tumor burden (number of tumors, tumor size, localization, and lymph node metastasis) in Step 2.

	Step 1					Step 2				
	B	[95% CI]		Beta	p	B	[95% CI]		Beta	p
		LL	UL				LL	UL		
Sex[a]	4.44	-1.31	10.19	.16	.128	6.69	.57	12.82	.24	.033
Age	.10	-.16	.36	.08	.463	.07	-.21	.34	.06	.612
Number						-.22	-.39	-.05	-.29	.010
Size						-.11	-.25	.04	-.16	.159
Localization[b]						3.93	-5.56	13.43	.09	.412
Metastasis[c]						.04	-6.30	6.38	.00	.990
	F(2, 88)=1,42, p=.247; R^2=.01					F(6, 75)=2,09, p=.064; R^2=.07				

[a]Female vs. male
[b]Leg vs. arm
[c]No vs. Yes

Table S3. Multiple and hierarchical ordinary least squares (OLS) regression showing the FACT-M subscale PWB score regressed on patient characteristics (sex, age) in Step 1, and tumor burden (number of tumors, tumor size, localization, and lymph node metastasis) in Step 2.

	Step 1					Step 2				
	B	[95% CI]		Beta	p	B	[95% CI]		Beta	p
		LL	UL				LL	UL		
Sex[a]	1.66	.13	3.19	.22	.034	2.40	.91	3.90	.31	.002
Age	.06	-.01	.13	.18	.074	.06	-.01	.12	.16	.098
Number						-.08	-.12	-.04	-.36	.000
Size						-.05	-.09	-.01	-.27	.009
Localization[b]						.90	-1.38	3.17	.08	.434
Metastasis[c]						.54	-1.01	2.10	.07	.489
	F(2, 91)=3,96, p=.022; R^2=.06					F(6, 78)=5,06, $p < .001$; R^2=.22				

[a]Female vs. male

[b]Leg vs. arm
[c]No vs. Yes

Table S4. Multiple and hierarchical ordinary least squares (OLS) regression showing the FACT-M subscale SWB score regressed on patient characteristics (sex, age) in Step 1, and tumor burden (number of tumors, tumor size, localization, and lymph node metastasis) in Step 2.

	Step 1					Step 2				
	B	[95% CI]		Beta	p	B	[95% CI]		Beta	p
		LL	UL				LL	UL		
Sex[a]	.03	-1.90	1.97		.972	-.27	-2.46	1.93	-.03	.809
Age	.01	-.08	.10		.829	-.00	-.10	.10	-.01	.961
Number						.01	-.05	.07	.06	.642
Size						-.01	-.06	.05	-.03	.822
Localization[b]						.46	-2.87	3.78	.03	.786
Metastasis[c]						.55	-1.73	2.82	.06	.633
	F(2, 90)=0,02, *p*=.976; R^2=.00					F(6, 77)=0,11, *p*=.995; R^2=.00				

[a]Female vs. male
[b]Leg vs. arm
[c]No vs. Yes

Table S5. Multiple and hierarchical ordinary least squares (OLS) regression showing the FACT-M subscale EWB score regressed on patient characteristics (sex, age) in Step 1, and tumor burden (number of tumors, tumor size, localization, and lymph node metastasis) in Step 2.

	Step 1					Step 2				
	B	[95% CI]		Beta	p	B	[95% CI]		Beta	p
		LL	UL				LL	UL		
Sex[a]	1.92	.12	3.73	.22	.037	2.56	.73	4.39	.29	.007
Age	.04	-.04	.12	.10	.313	.03	-.05	.11	.07	.498
Number						-.06	-.11	-.01	-.23	.029
Size						-.02	-.06	.03	-.09	.425
Localization[b]						1.91	-.99	4.81	.14	.194
Metastasis[c]						-1.39	-3.28	.51	-.15	.149
	F(2, 91)=2,71, *p*=.072; R^2=.04					F(6, 78)=2,87, *p*=.014; R^2=.12				

[a]Female vs. male
[b]Leg vs. arm
[c]No vs. Yes

Table S6. Multiple and hierarchical ordinary least squares (OLS) regression showing the FACT-M subscale FWB score regressed on patient characteristics (sex, age) in Step 1, and tumor burden (number of tumors, tumor size, localization, and lymph node metastasis) in Step 2.

	Step 1					Step 2				
	B	[95% CI]		Beta	p	B	[95% CI]		Beta	p
		LL	UL				LL	UL		
Sex[a]	1.37	-1.01	3.75	.12	.256	2.26	-.22	4.74	.19	.074
Age	-.03	-.14	.07	-.06	.547	-.03	-.14	.08	-.06	.594
Number						-.12	-.19	-.05	-.36	.001
Size						-.03	-.09	.03	-.11	.295
Localization[b]						.84	-3.11	4.78	.05	.674
Metastasis[c]						.02	-2.55	2.60	.00	.986

	F(2, 91)=0,85, p=.430; R^2=.00	F(6, 78)=2,61, p=.023; R^2=.10

[a]Female vs. male
[b]Leg vs. arm
[c]No vs. Yes

Table S7. Multiple and hierarchical ordinary least squares (OLS) regression showing the FACT-M subscale MS score regressed on patient characteristics (sex, age) in Step 1, and tumor burden (number of tumors, tumor size, localization, and lymph node metastasis) in Step 2.

	Step 1					Step 2				
	B	[95% CI]		Beta	p	B	[95% CI]		Beta	p
		LL	UL				LL	UL		
Sex[a]	2.23	-.80	5.25	.15	.147	3.80	.78	6.83	.25	.014
Age	.14	.00	.28	.21	.043	.16	.02	.30	.23	.023
Number						-.17	-.25	-.08	-.40	.000
Size						-.04	-.11	.04	-.10	.316
Localization[b]						2.04	-2.60	6.68	.09	.384
Metastasis[c]						.49	-2.65	3.64	.03	.757
	F(2, 92)=3,13, p=.048; R^2=.04					F(6, 79)=4,37, $p < .001$; R^2=.19				

[a]Female vs. male
[b]Leg vs. arm
[c]No vs. Yes

Table S8. Multiple and hierarchical ordinary least squares (OLS) regression showing the FACT-M subscale MSS score regressed on patient characteristics (sex, age) in Step 1, and tumor burden (number of tumors, tumor size, localization, and lymph node metastasis) in Step 2.

	Step 1					Step 2				
	B	[95% CI]		Beta	p	B	[95% CI]		Beta	p
		LL	UL				LL	UL		
Sex[a]	1.92	-.76	4.60	.14	.158	2.87	.35	5.39	.22	.026
Age	.22	.11	.34	.38	.000	.25	.14	.37	.43	.000
Number						-.09	-.15	-.02	-.24	.016
Size						-.06	-.12	.00	-.18	.070
Localization[b]						2.38	-1.35	6.11	.12	.207
Metastasis[c]						-1.98	-4.59	.63	-.14	.134
	F(2, 83)=8,35, $p < .001$; R^2=.15					F(6, 73)=6,74, $p < .001$; R^2=.30				

[a]Female vs. male
[b]Leg vs. arm
[c]No vs. Yes
